# The Medical Corporations and the West London Hospital

**Published:** 1915-08-07

**Authors:** 


### The Hospital

The Workers' Newspaper of Administrative Medicine and Institutional Life, Administration, National Insurance and Health.

No. 1521, Vol. LVIII. SATURDAY, AUGUST 7, 1915. PRICE ONE PENNY

### THE MEDICAL CORPORATIONS AND THE WEST LONDON HOSPITAL.

The difficulties of the voluntary hospitals are not diminishing. Their accommodation for the sick poor is seriously invaded by the War Office, which has not yet managed to organise a sufficient Hospital Service of its own for sick and wounded soldiers. In the circumstances, we admit, the soldiers ought not to be refused, but it is a painful task for boards of management to exclude the civil patients for whom the hospitals came into existence. The second continuing difficulty is the deficiency in the supply of resident medical officers. The reasons for this state of affairs are well known, and we need not repeat them. Expedients of various kinds have been suggested, but either they have not been adopted or they have not proved successful. Now, at the moment that the hospitals are crying out for young doctors, the Army is advancing the same invitation. Here, then, is an immediate problem for the medical world—to keep the hospitals going at their traditional level of efficiency and to meet the needs of the military authorities.

It is in these circumstances that the West London Hospital has addressed to certain of the medical corporations a request that fifth-year medical students acting as resident medical officers in the hospital should be allowed to count their term of service as a qualifying portion of the official medical curriculum. The effect of this proposal would be that a student having passed four or four and a half years at his medical school would complete his fifth year in the closest possible association with practical clinical work under the direction and control of experienced hospital physicians and surgeons. No one familiar with the conditions of medical education will question that such a scheme should mean for the student time spent to the best possible advantage, and this whether consideration be given to the practical demands of his final examination or to his equipment for the practice of his profession. Indeed, the claim may be made that the proposal is in strict harmony with the intention of the General Medical Council when adding a fifth year to the medical curriculum. The Council then recommended that this fifth year should be devoted to clinical work at a public hospital or dispensary, and though some medical schools and corporations may have set themselves to defeat this intention, it remains as the professed purpose of the extension of the curriculum. Even more conspicuous becomes the position when it is recognised that it is within the conditions of the Medical Council that six months of the curriculum may be passed by the student in attendance, quite apart from a hospital, on the practice of a private physician or surgeon. Yet when a hospital of whose staff and equipment there can be no question proposes that six months of the curriculum shall be passed in active work in its wards, the University of London and the Royal Colleges of Physicians and Surgeons refuse the request, while the strict legality of the scheme is made all the more decisive by the assent of the Society of Apothecaries.

From what we have already said it is evident that in our opinion the question can be argued on general grounds and altogether apart from the present emergency. But the proposal of the West London Hospital is limited to the duration of the war, and it may be well therefore to inquire how far it would help the existing position. In substance, and apart from any individual institution, the suggestion was advanced in our issue of May 8 as one method by which the present hospital difficulty could be relieved. If adopted, the immediate effect would be that a certain number of medical students would immediately take resident appointments in the voluntary hospitals. These institutions would therefore find officers, and this, too, at much smaller salaries than they are at present compelled to pay.

It may be asked, Would fifth-year students prove to be efficient officers? Of course, the admission must be made that in a technical and legal sense they would not be qualified medical practitioners. But will anyone argue that, judged by any practical test, there is a vital and essential difference between a student of four and a half years' standing and one who has just completed his fifth year and gained his diploma? Moreover, the choice at the moment is not between the advanced student and the newly qualified man. The latter, or the best part of him, has gone to the war, and it is his absence which is creating the difficulty.

The hospitals have often to fall back on men who for some reason or other are disengaged from practice, and many of whom have for years been out of contact with hospital methods. We do not hesitate to say that for efficient hospital work these are generally much less better prepared than are diligent students in the final year of their curriculum. Hence, keeping in view the present position only, we have no doubt that the admission of fifth-year students to resident appointments would improve over a wide area the existing conditions of hospital service. Possibly there may be public objection to the employment of students in responsible hospital duties. Here again we would plead the present emergency, and we would add that the objection is technical rather than substantial. Still further, resident officers act not independently but under the direction of seniors, and if these in the circumstances felt compelled to give a somewhat closer attention to their hospital duties the development would not be an altogether unhappy one. One word as to new doctors for the Army. It is allowed that no better preparation for active military service exists than some months' work as house surgeon in a well-organised hospital.

The proposal here discussed would increase the numbers of those trained in this fashion and would anticipate their training by some six months. Therefore it not only helps to meet the hospital emergency but also the Army emergency. That it has been rejected by the medical corporations is hardly surprising, seeing that these represent the medical schools, which are always ready to defend their monopoly and to tremble at the faintest suggestion of competition. These are not times for domestic struggles, but the action of the corporations at the present juncture will, we hope, not be forgotten.

